# Hippocampal Lesions Impair Rapid Learning of a Continuous Spatial Alternation Task

**DOI:** 10.1371/journal.pone.0005494

**Published:** 2009-05-08

**Authors:** Steve M. Kim, Loren M. Frank

**Affiliations:** 1 Neuroscience Graduate Program, University of California San Francisco, San Francisco, California, United States of America; 2 Department of Physiology, University of California San Francisco, San Francisco, California, United States of America; 3 W.M. Keck Foundation Center for Integrative Neuroscience, University of California San Francisco, San Francisco, California, United States of America; Max-Planck-Institut fuer Neurobiologie, Germany

## Abstract

The hippocampus is essential for the formation of memories for events, but the specific features of hippocampal neural activity that support memory formation are not yet understood. The ideal experiment to explore this issue would be to monitor changes in hippocampal neural coding throughout the entire learning process, as subjects acquire and use new episodic memories to guide behavior. Unfortunately, it is not clear whether established hippocampally-dependent learning paradigms are suitable for this kind of experiment. The goal of this study was to determine whether learning of the W-track continuous alternation task depends on the hippocampal formation. We tested six rats with NMDA lesions of the hippocampal formation and four sham-operated controls. Compared to controls, rats with hippocampal lesions made a significantly higher proportion of errors and took significantly longer to reach learning criterion. The effect of hippocampal lesion was not due to a deficit in locomotion or motivation, because rats with hippocampal lesions ran well on a linear track for food reward. Rats with hippocampal lesions also exhibited a pattern of perseverative errors during early task experience suggestive of an inability to suppress behaviors learned during pretraining on a linear track. Our findings establish the W-track continuous alternation task as a hippocampally-dependent learning paradigm which may be useful for identifying changes in the neural representation of spatial sequences and reward contingencies as rats learn and apply new task rules.

## Introduction

The hippocampal formation (comprising the dentate gyrus, CA3, CA2, CA1, subiculum, presubiculum, parasubiculum, and entorhinal cortex [1]) is essential for creating detailed new memories of experiences [2]–[5]. In non-human subjects such as laboratory rats, lesions of the hippocampal formation as well as non-destructive perturbations of hippocampal neural activity impair learning and memory in a variety of behavioral paradigms [6]–[14]. Parallel multielectrode single-unit recording studies in rats have revealed that neurons in the hippocampal formation code for diverse features of the rat's experience: past and present spatial locations in the environment, intended future destination of travel, running speed, head direction, landmarks, visual and geometric features of the environment, goal locations, odors, conditioned stimuli, and sequences of events [15]–[27]. Some studies have characterized changes in hippocampal neural coding during incidental learning upon changes in environment [28]–[34] and during task learning following a sudden change of task demands [35]–[39]. However, the functional contribution of the hippocampus to these forms of learning has not been established, so the significance of these neural correlates is unclear. We feel that it is important to acknowledge that the significance of neural coding phenomena in the hippocampal formation such as place cells, phase precession and sequential replay remains to be established. To date, no one has shown conclusively that any of these phenomena contributes to learned changes in behavior. Thus, while these various firing patterns clearly exist, and while there are hypotheses about their possible functional significance, we still lack a direct link between neural coding by hippocampal neurons and the learning and memory functions of the hippocampus.

Ideally, we would have a hippocampally-dependent learning paradigm that is suitable for single-unit recording studies. Unfortunately, most classic hippocampally-dependent learning paradigms are not suitable for investigating the learning-related dynamics of neural coding. In these learning paradigms, the subject is exposed to the task for only a few trials per day, and the behavior can be highly variable from trial to trial [40]–[42]. Because neurons are stochastic, accurate characterization of neural coding requires consistent sampling of behavior and spiking over many trials. As a result, it is difficult to characterize the relationship between neural activity and behavior in these classic learning paradigms. To overcome the disadvantages of undersampling and variability, investigators have designed hippocampally-dependent learning paradigms in which the behavior is carefully sampled over many repeated trials [43]–[50]. These recording-friendly learning paradigms are very useful, but learning of these tasks typically requires at least 6–7 days of training with 30–40 trials per day, depending on the exact learning criterion. Single-unit recording quality and yield tend to diminish over time, and it is difficult to maintain stable recordings of the same individual neurons across days. As a result, while these other established learning paradigms could potentially be used to study learning, it is not yet clear whether one could track neural dynamics within a single subject throughout the entire learning process.

We previously developed a W-track continuous alternation task that rats can learn quickly [17]. Using this task paradigm, we found that neurons in area CA1 of the hippocampus and in the entorhinal cortex exhibit task-relevant spatiotemporal coding, which (we speculate) could be used by other brain regions to guide task behavior. More recently, we found neural changes in the population-level distribution of firing rates in hippocampal area CA1 that paralleled behavioral changes in task performance [51]. At the same time, other investigators, using a similar but not identical maze-based continuous alternation task, observed that hippocampal neurons code for task-relevant spatiotemporal information even during performance of a task which can be accurately performed by rats with complete lesions of the hippocampus [52], [53]. This surprising observation suggests that the sensitivity of a task to hippocampal function is not necessarily correlated with task-relevant neural coding in the hippocampus. Here we investigated whether the learning of our W-track alternation task really depends on the hippocampal formation. We found that rats with extensive excitotoxic lesions of the hippocampal formation showed a dramatic deficit in acquisition of this task, whereas intact rats were able to learn this task in a few days. Thus, the W-track continuous alternation task may be a useful learning paradigm for investigating changes in neural representations that underlie memory formation and retrieval.

## Results

### Lesion evaluation

**Figure 1:**
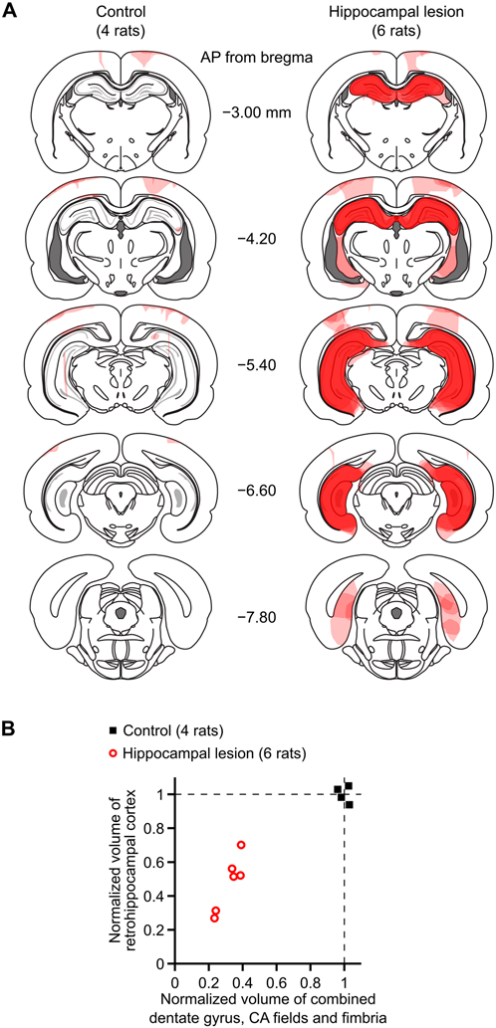
Histological reconstruction of hippocampal lesions. (A) Drawings of coronal sections at different anteroposterior levels illustrate the extent and location of brain damage, for subjects in the control group (left) and in the lesion group (right). Damaged areas within each subject are shaded in light pink; where there is overlap among subjects, the opacities of the overlapping regions sum to give darker shading. The darkest shade of red indicates areas that were consistently damaged in all subjects. The coronal section outlines are adapted from [76]. (B) Quantification of lesion extent. The horizontal axis is the estimated volume (combined over both hemispheres) of the dentate gyrus, CA fields, and fimbria. The vertical axis is the estimated volume (combined over both hemispheres) of the retrohippocampal cortex, which we define as the subiculum, presubiculum, parasubiculum, and entorhinal cortex. These volume estimates underrepresent the true loss of neurons because they include spared hippocampal white matter and partially-damaged shrunken tissue.

We tested 14 rats, of which 10 received hippocampal lesions and 4 underwent sham surgeries. We infused NMDA into the dentate gyrus, CA3, CA2, CA1, and subiculum (Table 1) to produce excitotoxic lesions of the hippocampal formation. At the end of behavioral testing, we sacrificed the rats and processed sections for Nissl staining and histological evaluation. Figure 1 shows the extent and location of damage for the subjects that were included in the final data analyses.

**Table 1:** Stereotaxic coordinates of NMDA infusions to produce complete lesions of the hippocampal formation.

AP (mm)	ML (mm)	DV (mm)
−2.8	±1.4	−3.0
−3.3	±2.4	−3.0
−4.1	±1.8	−2.8
−4.1	±3.4	−2.8
−4.8	±2.0	−2.8
−4.8	±4.2	−7.4
−4.8	±4.2	−3.1
−4.8	±5.0	−6.5
−5.5	±2.6	−3.0
−5.5	±3.6	−2.9
−5.5	±5.0	−7.0
−5.5	±5.0	−5.5
−5.5	±5.0	−3.5
−6.2	±4.0	−6.8
−6.2	±4.0	−3.4
−6.2	±5.4	−4.4
−6.8	±5.4	−4.0

The coordinates are given for a Long-Evans rat skull which is leveled so that bregma and dura lie in the same horizontal plane. AP, anteroposterior; ML, mediolateral; DV, dorsoventral. The anteroposterior and mediolateral coordinates are referenced to the skull at bregma, while the dorsoventral coordinates are distances below the dural surface.

All rats in the hippocampal lesion group sustained extensive loss of neurons in areas CA1, CA2, CA3, and the dentate gyrus (DG) throughout the entire longitudinal axis of the hippocampal formation. The neuropil was shrunken in these regions, and the ventricles had correspondingly expanded to fill the space. The alveus, fimbria, and hippocampal commissures were spared to various degrees in the lesioned rats. Hippocampally-lesioned rats also had variable damage to the subiculum, postsubiculum, presubiculum, parasubiculum and entorhinal cortex. Of the 10 rats with hippocampal lesions that we tested, 4 had extensive damage to regions outside of the hippocampal formation. These rats were removed from consideration, leaving 6 rats in the hippocampal lesion group (see Figure 1A for illustrations of lesion extent). We did include rats that had either (1) circumscribed damage to the white matter and visual/parietal cortex dorsal to the hippocampus, or (2) circumscribed damage to thalamic nuclei adjacent to the hippocampus. To quantify the lesions, we measured the total volume of remaining tissue within the dentate gyrus and CA fields (including the adjacent fimbria), as well as the total volume of remaining tissue in retrohippocampal structures (subiculum, presubiculum and parasubiculum and entorhinal cortex). These reconstructed volumes, normalized with respect to the mean of the control group, are plotted in Figure 1B. Note that these total tissue volumes underrepresent the true loss of hippocampal neurons, because they include intact white matter and partially-damaged areas in which the density of neurons was severely reduced.

In all of the behavioral results that we report below, we found no obvious correlation between lesion extent and variability of task behavior. Subjects in the sham-surgery control group (4 rats) sustained variable amounts of damage to the visual/parietal neocortex and to the white matter overlying the hippocampus. The hippocampal formation was intact in all of these rats.

### Linear-track running task

**Figure 2:**
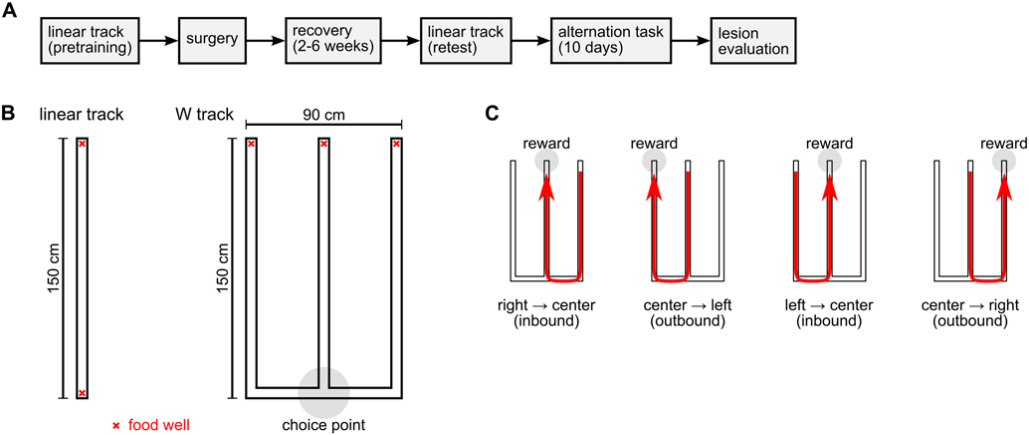
Experimental design and behavioral tasks. (A) Timeline of the experiment. (B) Diagrams of the running tracks used in this experiment. The red X marks indicate locations of food wells. The gray circle indicates the choice-point intersection on the W track. (C) Sequential illustration of correct performance of the W-track continuous spatial alternation task. Rats were rewarded for visiting the three food wells of the W track in the correct repeating sequence.

Before surgery, we trained rats to shuttle back and forth between food wells located at the two ends of a linear track (Figure 2A,B). Rats had to run the entire length of the track to receive food reward; no reward was given for consecutive repeat visits to the same food well. All rats were trained to the same performance criterion. After recovery from surgery, we tested the rats again on the same linear track. We compared task performance on the last day of pre-surgery training and at the post-surgery test so that we could account for possible confounding effects of hippocampal lesion on locomotion or food-seeking.

To quantify performance on the linear track, we parsed the running behavior into a sequence of “trials”. Note that during the actual task behavior, rats transitioned between successive trials without interruption, and no trial-timing cues were provided. We defined the start of a trial by the rat's departure from a food well, and likewise we defined the end of trial by the rat's next arrival at a food well. A correct trial started with departure from one food well and ended with arrival at the opposite food well, whereas an incorrect trial was one on which the rat prematurely returned to the same food well from whence it had departed. For each rat, we computed the proportion of trials that were correct; the total number of trials completed; mean running speed while in transit between food wells; and the median duration of the end-of-trial food-well visit. We used nonparametric repeated-measures tests [54] for statistical comparisons between the hippocampal lesion group and the control group.

**Figure 3:**
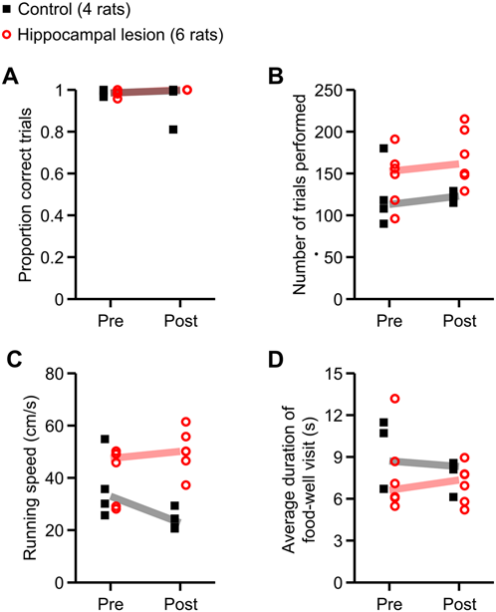
Summary of performance on linear track task. Plotted in each panel is a measure of linear track behavior before surgery (Pre) and after recovery from surgery (Post). Filled black symbols indicate values for the control group (4 rats), and open red symbols indicate the same for the hippocampal lesion group (6 rats). The correspondingly color-coded heavy lines are group medians. (A) Proportion of correct trials. (B) Number of trials completed. (C) Mean running speed, excluding times spent at food wells. (D) Median dwell time at food wells at the end of trials. Only running speed significantly differed between the groups (main effect of group, *p* = 0.0092; group×day interaction, *p* = 0.012). *Post hoc* within-day comparisons revealed that the difference in running speed between the two groups was not significant on the last day of pre-surgery training (*p* = 0.76), but was significant for the post-surgery test (*p* = 0.0095). Thus, hippocampal lesions caused an increase in running speed but did not disrupt task performance on the linear track.

There were no significant between-group differences in the proportion of trials performed correctly, the total number of trials completed, or the median food-well visit duration (Figure 3A–C). However, running speed (Figure 3D) showed a significant main effect of group (*p* = 0.0092) as well as a significant group×day interaction (*p* = 0.012). To examine the temporal pattern of this effect, we did within-day pairwise *post hoc* comparisons using the Wilcoxon rank-sum test. The difference in running speed between the two groups did not reach statistical significance on the last day of pre-surgery training (*p* = 0.76), but was significant for the post-surgery test (*p* = 0.0095). Thus, hippocampal lesions caused an increase in running speed but did not disrupt the fluency of task performance on the linear track.

### W-track continuous spatial alternation task

We introduced the rats to the W-track continuous spatial alternation task on the day after the post-surgery test on the linear track (Figure 2B,C). We tested the rats on this task for 10 consecutive days. Rats had no prior experience with the W track. At the beginning of each session, each rat was simply placed on the center arm of the W track and allowed to explore uninterruptedly. The food wells at the ends of the three arms dispensed fixed reward according to the following rules: (1) A visit to the center food well was rewarded when the rat came from either side food well. (2) A visit to the left or right food well was rewarded when the rat came from the center food well after having previously visited the opposite side food well. (3) Consecutive repeat visits to the same food well were never rewarded. Together, these rules defined a correct cyclical sequence of food-well visits (Figure 2C): right, center, left, center, right, center, left, center, etc.

The correct task sequence on the W track can be decomposed into two interleaved components. When the rat departed from the left food well or from the right food well, the correct destination was always the center food well. We use the term “inbound” to describe this return-to-center component of the task. In contrast, when the rat departed from the center food well, it needed to remember which side of the W track it had last come from, because the correct destination was the opposite-side food well. We use the term “outbound” to describe this side-alternation component of the task. Note that the inbound and outbound task components correspond respectively to “reference” and “working” memory, as classically defined [55].

**Figure 4:**
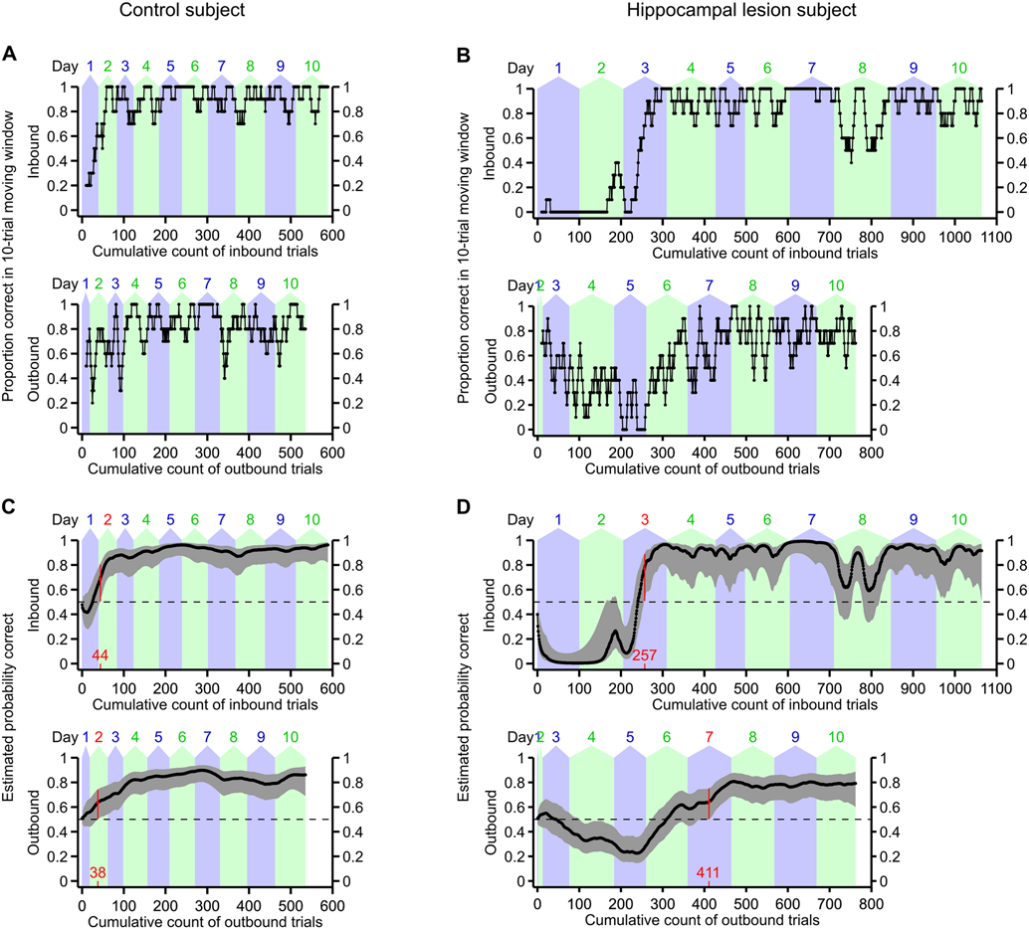
Examples of learning curves on the W-track continuous alternation task for two subjects. (A) 10-trial moving average of proportion correct for a control subject. The top plot shows performance on inbound trials, while the bottom plot shows performance on outbound trials. Trials are counted cumulatively along the horizontal axis, starting with the first trial on day 1 and ending with the last trial on day 10. The alternating blue and green background shading indicates the number of trials completed on each day. (B) 10-trial moving average of proportion correct for a lesion subject. (C) Smooth learning curve estimated using the state-space model of learning, for the same subject as in (A). The top plot shows the estimated learning curve for the inbound component of the task, while the bottom plot shows the estimated learning curve for the outbound component of the task. Trials are counted cumulatively along the horizontal axis in the same manner as in (A). Black dots indicate maximum-likelihood estimates of the probability of correct performance, and gray errors bars indicate point-wise 95% confidence intervals. Dashed horizontal lines indicate the chance performance level (1/2) that would be expected if the subject randomly chose the next destination food well. We defined the learning criterion (highlighted in red) as the earliest trial at which the 95% confidence interval of the learning curve exceeded this chance level and remained above chance for two full consecutive days. (D) Similar to (C), but for the same hippocampal lesion subject as in (B). The initial low dip of the inbound learning curve, and the paucity of outbound trials, reflects the many perseverative inbound errors that this subject made during the first two days of testing. This subject's peformance on the inbound component of the task regressed transiently on day 8 for unknown reasons.

To quantify performance of the W-track continuous alternation task, we parsed the running behavior into trials and classified the trials as inbound or outbound according to their point of origin on the W track. All trials in which the rat departed either from the left food well or from the right food well were classified as inbound trials, and all trials in which the rat departed from the center food well were classified as outbound trials. Examples of 10-trial moving averages of task performance, separated by inbound versus outbound trials, are shown for one control subject and one hippocampal lesion subject in Figure 4A and B. (Moving-average plots for all subjects are shown in Figure S1 and Figure S2.) While this sort of moving average is frequently used to evaluate behavioral performance, it is difficult to compute meaningful confidence bounds for individual animals using this analysis. We therefore used a state-space model of learning [56] to estimate individual learning curves for each subject on both the inbound and outbound components of the W-track alternation task (see Materials and Methods for details). This model uses the observed data to estimate the subject's probability of making a correct choice from trial to trial, along with confidence bounds on that estimated probability. This state-space model-based analysis has a number of advantages over moving average or change-point analyses, including the ability to estimate confidence bounds for individual subjects and greater sensitivity to changes associated with learning [56]. Examples of learning curves for one control subject and one hippocampal lesion subject are shown in Figure 4C and D. (Learning curves for all subjects are shown in Figure S3 and Figure S4). These learning curves are estimates of the probability of correct performance, with 95% confidence intervals, as a function of the number of trials completed by the subject.

We used these smooth estimated learning curves to quantify how quickly the subjects learned the inbound and outbound components of the W-track continuous alternation task. Specifically, we identified the first trial and test day on which the 95% confidence interval of the estimated probability of correct performance exceeded and remained above chance throughout at least two full consecutive days of testing. This learning criterion has an intuitive interpretation as earliest timepoint at which we can be reasonably certain that the subject is capable of performing the task at consistently above-chance level for a sustained duration. Furthermore, this earliest-timepoint learning criterion is robust to temporary lapses in task performance which sometimes occur even after a subject has exhibited fluent task performance. Because the W-track continuous alternation task is not a forced-choice task paradigm, it is somewhat debatable what exactly constitutes “chance” performance. However, we reasoned that, starting for any given food well, the rat could proceed to either of the two other food wells on the W track, which yields a chance probability of 1/2. This chance level is indicated by the dashed horizontal lines in Figure 4C and D. In fact, the rats were free to also revisit the same food well from whence they had just departed, so we also performed this analysis with respect to a 1/3 chance probability. We obtained qualitatively similar results with both chance levels, but because the 1/2 chance probability corresponds to a more stringent learning criterion, we chose to present the results using that criterion.

**Figure 5:**
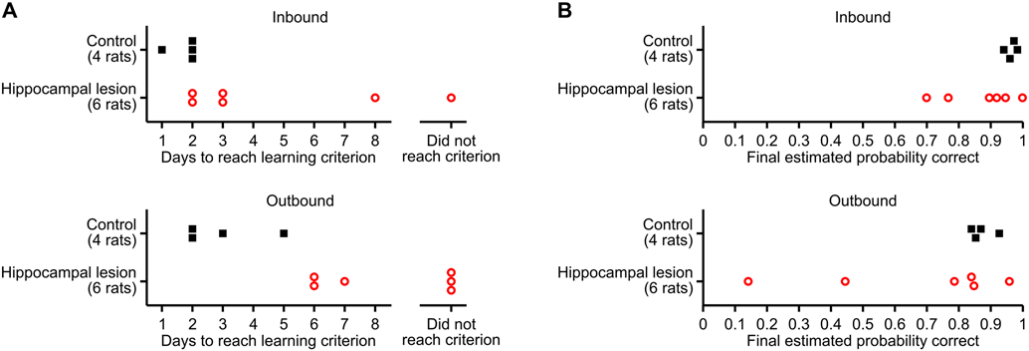
Effect of hippocampal lesions on learning of the W-track continuous spatial alternation task. (A) Number of days to reach learning criterion on the inbound (top) and outbound (bottom) components of the task. Compared to the control group, the hippocampal lesion group exhibited significantly slower learning of the outbound task component (*p* = 0.0095). (B) Mean estimated probability of correct performance on day 10 of testing. Although there is an apparent trend for the performance of the hippocampal lesion subjects to be skewed lower, this trend did not reach significance.

All control rats reached the learning criterion on both the inbound and outbound components of the W-track continuous alternation task within a few days. In contrast, 1 out of 6 lesion subjects failed to reach the learning criterion on the inbound task component, and 3 out of 6 lesion subjects failed to reach the learning criterion on the outbound task component (Figure 5A; Table S1). We used the Wilcoxon rank-sum test to compare the number of trials/days to reach learning criterion between groups. For those subjects that did not reach the learning criterion by the end of testing, we imputed the learning trial to be the earliest trial on which performance exceeded chance level and remained above chance over all remaining observed trials, or if even this relaxed criterion was not satisfied, we used 1+[total number of trials completed]. These conservative imputations of the truncated learning curves allowed us to include all subjects in the statistical tests. Compared to control rats, rats with hippocampal lesions required a greater number of inbound trials to reach learning criterion on the inbound component of the task (*p* = 0.019), and they also required a greater number of outbound trials to reach learning criterion on the outbound component (*p* = 0.0095). When we analyzed the number of test days to reach learning criterion, the learning impairment of the lesion group was statistically significant on the outbound component of the task (*p* = 0.0095), but the trend on the inbound component of the task did not reach statistical significance (*p* = 0.095). We also compared final task performance on day 10 between the two groups, and found that the groups did not significantly differ on either inbound or outbound trials, although there appears to be a trend for the lesion group to be skewed towards poorer task performance (Figure 5B; Table S1). Together, these results suggest that hippocampal lesions retard learning of the W-track continuous alternation task but do not prevent eventual fluent task performance.

**Figure 6:**
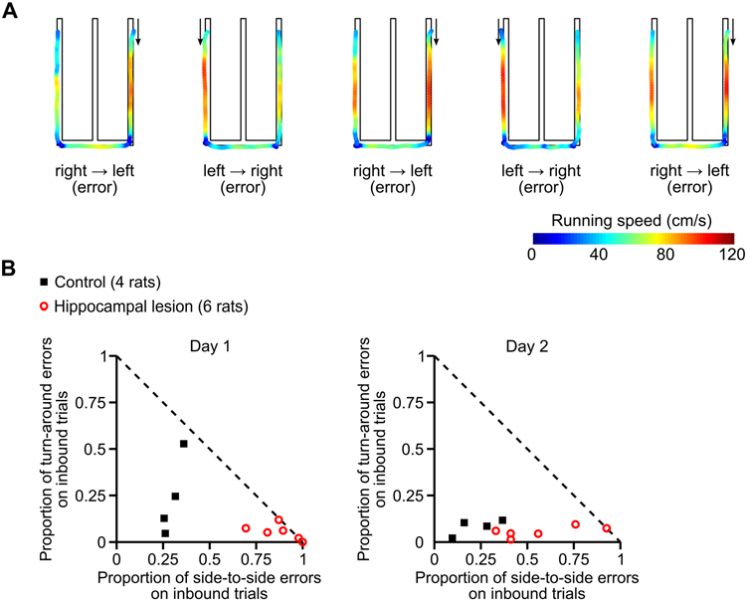
Effect of hippocampal lesions on inbound errors during early task experience. (A) Example of perseverative errors made by a hippocampal lesion subject during the first session of the W-track continuous alternation task. Path-maps are shown for five consecutive incorrect inbound trials. The paths are color-coded to indicate the rat's instantaneous running speed. Arrows indicate the direction of travel. (B) Scatterplots showing the pattern of inbound errors on on day 1 (left) and day 2 (right) of the W-track continuous alternation task. The plotted symbols show, for each individual subject, the proportions of errors on inbound trials, classified according to destination: the proportion of inbound trials in which the subject ran from one side food-well to the opposite side, skipping the center arm (horizontal axis); and the proportion of inbound trials in which the subject returned to the outside arm food-well from which it had just departed (vertical axis). The dashed diagonal line indicates the maximum possible values of these error proportions. A larger proportion of lesioned animals' inbound trials were associated with side-to-side trajectories on both day 1 and day 2 as compared to controls (day 1, *p*<0.01; day 2, *p*<0.04).

We found another difference between the groups on inbound trials during the first two days of experience on the W track. On the first day of testing, all six of the lesioned animals performed well below chance levels on inbound trials, while only 1 out of 4 of the control animals showed a comparable tendency. The dramatically below-chance performance of the hippocampal lesion subjects suggested some initial bias or perseveration. We inspected the recorded video of the behavior and noticed that some rats had a tendency to repeatedly run from one side of the W track to the opposite side, entirely skipping the center arm (Figure 6A). To quantify this tendency, we classified errors on inbound trials according to rat's choice of destination: trials on which the rat ran from one side food-well to the opposite side (while skipping the center arm) were classified as side-to-side errors; trials on which the rat returned to the outside arm food-well from which it had just departed were classified as turn-around errors. We found that the control group and the hippocampal group committed these two types of inbound errors in different proportions (Figure 6B,C). Specifically, lesioned animals committed a larger proportion of side-to-side errors on inbound trials on both day 1 and day 2 (Wilcoxon rank sum test: day 1, *p*<0.01; day 2, *p*<0.04). Thus, these animals perseverated in running from one side food well to the opposite side food well, even though that trajectory was never rewarded.

This early perseverative behavior appeared to contribute to the larger number of trials required for the lesioned animals to learn the inbound component of the task. Indeed, a visual inspection of Figure 6B and C along with the learning curves in Figure S4, suggested that once the lesioned animals reduced their perseverative, below-chance behavior, their performance rapidly increased. We attempted to measure the slopes of the learning curves either following that initial perseverative behavior or around the learning trial, but we were unable to construct a measure that could be applied consistently and sensibly to all lesioned animals due to the variability in their performance. Thus, we did not feel that we could effectively quantify the rate of post-perseverative learning.

**Figure 7:**
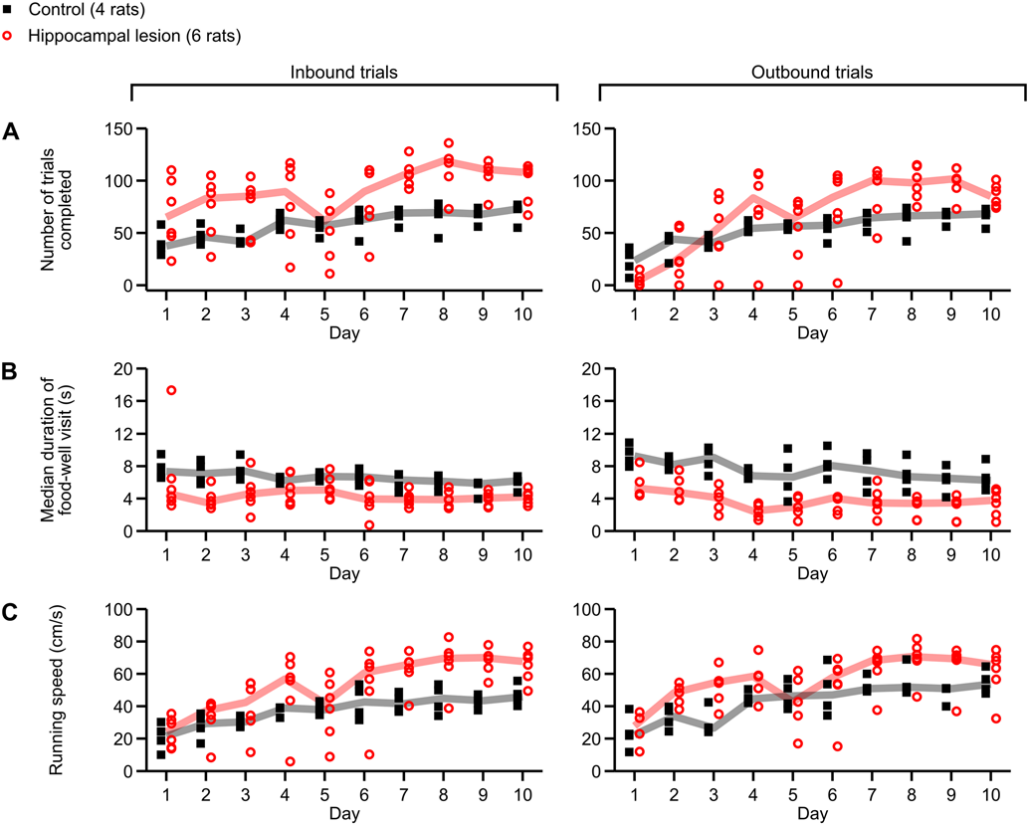
Effect of hippocampal lesions on behavior on the W track. Plotted in each panel is a measure of behavior across all 10 days of testing on the W track. Filled black symbols indicate values for the control group (4 rats), and open red symbols indicate the same for the hippocampal lesion group (6 rats). The correspondingly color-coded heavy lines are group medians. (A) Number of inbound (left) and outbound (right) trials performed on each day of testing. Compared to control subjects, subjects with hippocampal lesions tended to perform more inbound trials (*p*<0.02). (B) Average dwell time per food-well visit at the end of inbound (left) and outbound (right) trials. Compared to lesioned subjects, control subjects tended to dwell at the food well for a longer time after each trial (inbound, *p*<0.005; outbound, *p*<0.002), in part because they completed a greater proportion of trials correctly and thus spent more time consuming food reward. (C) Running speeds on inbound and outbound trials. Compared to control subjects, lesioned subjects ran at higher speeds on both inbound (*p*<0.05) and outbound (*p*<0.04) trials.

To rule out the possibility that rats with hippocampal lesions had nonspecific impairments of locomotion or motivation, we examined the number of trials performed, food-well dwell times and running speeds for inbound and outbound trials (Figure 7). We found that lesioned animals tended to perform more inbound trials than controls (main effect of group, *p*<0.02). While there was no significant difference in the number of outbound trials performed, there was a significant (*p*<0.005) group×day interaction. This interaction can be seen by the fact that the lesion group tended to perform fewer outbound trials than the control group during initial task experience (day 1), but tended to perform more outbound trials than the control group during later task experience (days 7–10). The deficit in the number of outbound trials performed by lesion subjects on day 1 can be ascribed to their early perseverative failure to visit the center arm on inbound trials, as previously shown in Figure 6. The poorer overall task performance of the lesion group was also accompanied by decrease in food-well dwell times on both inbound (main effect of group, *p*<0.005) and outbound (main effect of group, *p*<0.002) trials. This difference was directly related to the better performance of the control group, as animals paused longer at the wells when they were rewarded. When we computed food-well visit duration separately for correct trials and for error trials, the differences between groups were no longer statistically significant (data not shown). Running speed was similarly different between groups, with higher running speeds for the lesioned group on both inbound (*p*<0.05) and outbound (*p*<0.04) trials. The ample numbers of trials that were completed and the fast running speeds indicate that rats with hippocampal lesions did not lack motivation or locomotor drive.

## Discussion

We found that rats with lesions of the hippocampal formation are significantly impaired at learning the W-track continuous spatial alternation task. Compared to control rats that had undergone sham surgeries, rats with extensive lesions of the hippocampal formation made more errors and took longer to reach learning criterion on both the inbound (reference memory) and outbound (working memory) components of the task. These effects could not be attributed to lesion-induced deficits in locomotion or food-seeking motivation, because rats with hippocampal lesions successfully performed the linear-track running task. We did observe that, compared to control rats, rats with hippocampal lesions ran faster and completed more trials on the linear track and during the latter days of testing on the W track. These results are consistent with previous reports that hippocampal lesions can cause locomotor hyperactivity [57]–[62]. However, hyperactivity cannot explain why the rats with hippocampal lesions exhibited such a dramatic pattern of perseverative errors on inbound trials during initial task experience.

The W-track continuous alternation task is similar to other maze-based running tests of hippocampal function, such as the radial maze working-memory task [13] and the delayed continuous T-maze alternation task [53]. A distinguishing feature of this task is that working-memory and reference-memory demands are regularly interleaved between trials, and the time in the center arm serves as a built-in “delay” period. We found that lesions of the hippocampal formation impaired learning of both mnemonic components of the task. However, some of the subjects with hippocampal lesions were able to acquire both the inbound and outbound components of the task by the end of the 10-day test sequence. Thus, given enough prolonged experience, other brain regions can support the learning of this task even in the absence of a functional hippocampus. The lengths of time required for lesioned animals to learn the outbound component of the task correspond reasonably well to the amounts of time required for animals to switch from a hippocampal to a basal ganglia dependent strategy in a plus-maze task [63]. Thus, we speculate that plasticity in circuits associated with the basal ganglia may support the slower learning seen in the lesioned animals. It is not known whether the transition from hippocampal control to extra-hippocampal control involves intrinsic changes within the hippocampal formation, or if instead it involves some complex gating of hippocampal output in coordination with other regions. We think that recording experiments to probe those possibilities would be very informative.

In our experiment, we challenged rats to learn a complex task sequence through trial-and-error exploration of an unfamiliar environment. On outbound trials, there was no simple sensory stimulus that predicted reward. Instead, alternation between side arms required internal representation of the recent history of trials to the side arms. Our finding of an effect of hippocampal damage on this type of learning is consistent with the theory that the hippocampal formation is important for incremental learning of the latent structure of the world, such as stimulus regularities and environmental context [64]. It also agrees with theories that the hippocampal formation supports reinforcement learning of the paths that lead to reward. During learning of the alternation task, the rat must integrate reward information with memory of the recently-visited sequence of food wells [65]–[67].

Our observation that rats with hippocampal lesions perform perseverative side-to-side errors on inbound trials is reminiscent of previous reports that hippocampal lesions result in perseverative failure to suppress conditioned behavioral responses when reward is diminished or when reward contingencies are switched [68]–[72]. We speculate that this perseverative tendency may be a consequence of the pretraining procedure, in which we trained rats to run from end to end along the entire length of a linear track. In the pretraining situation, uninterrupted running behavior led to maximum exploitation of available food rewards. We speculate that rats with hippocampal lesions transferred their previously acquired habitual responses to the W track, instead of exploring and re-optimizing their behavior according to the new task rules. Thus, the effect of hippocampal lesions on performance of the inbound, reference memory portion of the task could have resulted from a requirement for the hippocampus in inhibiting a previously acquired response. If this explanation is true, then the prediction is that the perseverative side-to-side errors will be less severe if rats are not pretrained to shuttle back and forth on a linear track. This prediction remains to be tested in future studies.

This study enhances the significance of previous recording studies. In a previous study, we recorded the activity of neurons in the hippocampus and in the entorhinal cortex while well-trained rats performed the same W-track alternation task fluently; we found that neurons in these areas exhibit trial-specific coding for prospective destinations on outbound trials and retrospective origins on inbound trials [17]. Thus, there is an intriguing correspondence between task demands and neural activity. Our results here indicate that animals can learn this task without the hippocampus, but that learning is slower in that case. We speculate that prospective and retrospective coding by hippocampal neurons may contribute to early, rapid learning but is not necessary for later fluent performance of the task. This possibility is consistent with the observation that hippocampal neurons exhibit such spatiotemporal coding even during task behavior that does not depend on the hippocampus [53]. Thus, future experiments will need to determine whether these trial-specific coding emerges in parallel with time course of learning.

We have also found that changes in the distribution of firing rates over the population of place cells in hippocampal area CA1 occur on the same timescale as changes in behavioral performance during learning of the W-track continuous alternation task [51]. In this study, we found that firing rates and spatial specificity of neurons in hippocampal area CA1 are plastic across the first 5–6 days of task experience and subsequently stabilize. If this neural coding plasticity in the hippocampus is required for learning during the first few days of task experience, we would expect that rats with hippocampal lesions would be unlikely to learn the entire task (outbound and inbound) in less than 6 days; indeed, in this study, we found that the three lesioned animals that managed to master the outbound portion of the tasks did so on days 6, 7 and 9. Our demonstration here that learning of this task is sensitive to hippocampal damage strengthens the hypothesized connection between changes in hippocampal neural activity and learning in this task, and provides a foundation for future studies.

Finally, efforts to develop behavioral tasks that are both dependent on an intact hippocampus and suitable for electrophysiology may facilitate new studies that link specific patterns of hippocampal neural activity to behavioral change. We know quite a lot about neural coding phenomena exhibited by hippocampal neurons during both waking and sleep [e.g. 73,74,75], but most of these studies used open field random foraging or linear track alternation tasks which did not impose demands on hippocampally-dependent learning or memory. Given that an intact hippocampus is not essential for behavior in these tasks, it remains possible that these neural coding phenomena are not directly related to the learning and memory functions of the hippocampus. Understanding how hippocampal neural activity and plasticity underlie hippocampally-dependent learning thus requires the use of tasks that demonstrably engage and require hippocampal circuitry. In conjunction with targeted manipulations of neural activity or plasticity at specific timepoints during learning, we believe that the hippocampally-dependent W-track continuous alternation task is well suited to help us link neural coding with behavior.

## Materials and Methods

### Subjects

We used 26 male Long-Evans rats obtained from a commercial breeder (Taconic Farms). Rats were singly housed in polycarbonate cages (42×21×21 cm) with recycled paper pellet bedding and *ad libitum* access to drinking water. Temperature, humidity and illumination (12:12-hour light/dark cycle) in the housing facility were artificially controlled. Behavioral testing occurred during the lights-on phase. All procedures were approved by the Institutional Animal Care and Use Committee at the University of California, San Francisco.

### Training before surgery

Upon arrival in the housing facility, rats had *ad libitum* access to standard laboratory rat chow pellets. We gradually habituated the rats to daily human handling over several weeks. After every handling session, each rat was given access to a licking spout in his home cage that delivered evaporated milk (Carnation brand, Nestlé) sweetened with 0.2% saccharin (Smoky Mountain Sweetener). This procedure guaranteed that the rats overcame their food neophobia to recognize the palatability of this liquid food reward.

After habituation, feeding was restricted to maintain the rats at 85–88% of their baseline free-feeding body mass, as verified by daily weighings. We trained the rats to run back and forth along an elevated linear track (150 cm long, 6 cm wide). Rats were motivated with droplets of sweetened milk, which were automatically dispensed in food wells located at the two ends of the track. Rats received a fixed amount of food reward on every visit to a food well, except that no reward was given for any consecutive repeat visits to the same food well. A monochrome CCD camera mounted above the linear track captured video of the rat's behavior (30 frames per second at 320×240 resolution), which was streamed to the NSpike data acquisition system (L.M. Frank, J.MacArthur) and processed for automated delivery of food reward. The linear track and the floor were colored bright white, so that the dark pigmented hood and stripe of the Long-Evans rats could be identified in video images simply by luminance contrast.

Rats were trained on the linear track for two 15-minute sessions per day. Each rat finished training and underwent surgery after he performed at least 30 correct food-well visits per session, in all four sessions over two consecutive days. We removed 3 rats from the cohort of subjects before surgery because they failed to reach this performance criterion after 7 days of training.

### Surgery

Rats were 3–5 months old at the time of surgery. Each rat was randomly assigned to either the control group or the hippocampal lesion group; this group assignment was blind to individual performance on the linear track. General anesthesia was induced with 5% isoflurane in oxygen and maintained with 1–5% isoflurane. We administered atropine (0.04 mg/kg, i.p.) to reduce airway secretions and buprenorphine (0.04 mg/kg, i.p.) for analgesia. We secured the anesthetized rat in a stereotaxic head frame (David Kopf Instruments) with a thermostat-regulated heating pad (37°C) to prevent hypothermia. After exposing the skull, we adjusted the height of the incisor bar so that bregma and lambda were in the same horizontal plane and then drilled craniotomy in the skull overlying the hippocampus. We made bilateral excitotoxic lesions of the hippocampal formation by infusing NMDA (Sigma) dissolved in artificial CSF (20 mg/mL) at 17 sites in each hemisphere (listed in Table 1), with the intention of targeting the dentate gyrus, CA fields, and subiculum. We made the infusions with a 26 ga microliter syringe-needle (Hamilton) mounted in a motorized syringe driver (KD Scientific), which was attached to an arm of the stereotaxic frame. At each site, we infused 0.08 µL of NMDA solution at a rate of 0.10 µL/min, and then waited 2 minutes after the end of infusion before retracting the needle. During sham surgeries, we filled the Hamilton syringe with the NMDA solution and positioned the needle in the brain at the same locations for the same durations, but the syringe plunger was not driven to effect infusion. This sham procedure was intended to control for extraneous damage during passage of the NMDA-loaded needle on the way to the hippocampus. We administered diazepam (10 mg/kg) intraperitoneally before the cessation of general anesthesia as a prophylactic against seizures; subjects in the control group also received diazepam. After surgery, we administered meloxicam (0.4 mg/kg) subcutaneously every 18 hours for analgesia until full healthy recovery from surgery. Nine rats died during surgery or were euthanized because of poor recovery after surgery. We believe that this high mortality rate was due to our relative inexperience with these techniques, combined with our efforts to produce complete lesions.

### Testing after surgery

After 2–6 weeks of recovery from surgery, restricted feeding was resumed. The rats were tested again on the familiar linear track for two 15-minute sessions, to control for any possible effects of surgery on food-seeking motivation or locomotion. Next, they were tested on the W-track continuous spatial alternation task for 10 days, in two 15-minute sessions per day. We did not give the rats any prior habituation or shaping on the W track. Sweetened milk was automatically dispensed in food wells located at the three ends of the track, according to the following rules: (1) A visit to the center food well was rewarded when the rat came from either side food well. (2) A visit to the left or right food well was rewarded when the rat came from the center food well after having previously visited the opposite side food well. (3) Consecutive repeat visits to the same food well were never rewarded. The rats were free to choose any of 3^2^ = 9 possible combinations of start/end points for their journeys on the W track. At the beginning of each session, the experimenter placed the rat on the center arm facing the center food well, which was pre-baited with sweetened milk. Because of this initial task state, scoring of the first outbound trial (i.e., following departure from the center food well) was ambiguous with respect to the left/right alternation rule. To avoid confusing the rats, we always rewarded the first visit to a side food well within a session, but we did not include this first outbound trial when analyzing task performance.

### Histology

At the end of behavioral testing, we killed the rats with an overdose of Euthasol (Virbac) and perfused transcardially with isotonic sucrose followed by 4% formaldehyde in phosphate-buffered buffered saline. After overnight post-fixation in 4% formaldehyde, the brains were stored in a cryoprotectant solution (20% glycerol/2% DMSO). Frozen brains were sectioned coronally (50 µm) on a cryostat microtome, starting from the caudal pole of the cortex and continuing rostrally throughout the entire extent of the hippocampal formation. Every other section was mounted on a gelatin-coated glass slide and stained with cresyl violet to visualize Nissl substance. We captured brightfield photomicrographs at 20× magnification, using a Photometric Coolsnap HQ2 camera attached to a Nikon TE2000E microscope. Photomicrographs were merged into whole-section montage images using Nikon NIS-Elements software. We examined the pattern of Nissl staining with reference to published anatomical guides [1], [76] in order to evaluate the lesions. We quantified lesion extent with the Cavalieri method [77]. We measured the volume of intact hippocampal tissue within both hemispheres from cross-sectional outlines taken at every 200 µm throughout the anteroposterior extent of the hippocampal formation.

### Analysis of behavior

We processed the video data to estimate the rats' movement trajectories along the two running tracks. In each video frame, we identified pixels whose grayscale luminance values were less than a certain threshold. The largest contiguous cluster of these dark pixels corresponded to the pigmented fur (“hood”) of the rat, which was clearly visible against the white background surfaces. We tracked the centroid of this pixel cluster in every video frame, and then applied nonlinear smoothing (denoising) to the sequence of centroids [78] to estimate the position of the rat. To estimate instantaneous velocity, we took first-order differences of the position estimate and multiplied by the video frame rate. We converted pixel distances to physical distances (centimeters) for these measures.

Next, we marked the locations of food wells in the video images and defined corresponding regions of interest (2 ROIs on the linear track, 3 ROIs on the W track) centered on these locations. Each ROI was a circle with a 15-cm radius. Using the estimated movement trajectories, we determined the times when the rat entered or exited the ROIs. These transition times were used to reconstruct the sequence of food-well visits and the durations of those visits. Inbound and outbound trials were automatically scored according to the rules for the task.

### Estimation of learning curves

We used a state-space model of learning [56] to estimate individual learning curves on the W-track continuous alternation task. This model describes an animal's choice behavior as a evolving process. At each trial, the model estimates the value of a hidden (e.g. not directly observable) “state” variable that represents the probability of making a correct choice. The model simultaneously estimates confidence bounds for the state variable, representing the level of uncertainty about the probability of a correct choice. We used the expectation maximization algorithm to find the set of values that best describe the animal's choice behavior across time. The result is a more accurate estimate of learning-related changes in choice behavior than arises from standard moving average or choice-point measures of learning [56].

Mathematically, the observed task responses are treated as outcomes of a Bernoulli process whose success rate (i.e., the probability of correct performance on each trial) is linked to a hidden learning state. The evolution of the hidden learning state is modeled as a Gaussian random walk of unknown variance. Given the observed outcomes of an experiment, the hidden learning state can be estimated with some uncertainty; the principled treatment of uncertainty in the state-space model provides advantages over alternative constructions of learning curves (e.g., moving average).

For simplicity, we estimated separate learning curves for the inbound and outbound components of the W-track continuous alternation task. We chose this simplification because we could not parsimoniously model the statistical dependence between the two task components. To estimate a learning curve for the inbound component of the task, we considered the outcomes (correct versus incorrect) of all trials that departed from either the left food well or the right food well. These outcomes were concatenated into a single long sequence that spanned the subject's entire task experience on the W track. Similarly, we concatenated the outcomes of all trials that departed from the center food well to estimate a learning curve for the outbound component of the task. As described in [56], we then estimated (with confidence intervals) the evolution of the hidden learning state from the sequence of observed outcomes. This algorithm required an initial proposal for the baseline probability of correct performance. We set this chance probability at 1/2, reasoning that the subjects would initially choose randomly one of the two other food wells as a destination when departing from a food well. To confirm that the results were not overly sensitive to this initial proposal probability, we also estimated the learning curves with the chance probability set to 1/3, which corresponds to random equiprobable choice from among all three food wells on the W track.

## Supporting Information

Table S1 Summary statistics for individual subjects on the W-track continuous alternation task. Each column corresponds to an individual subject; C1-C4 are control subjects, and L1-L6 are hippocampal lesion subjects. The p-value column shows the result of the Wilcoxon rank-sum comparison between the two groups. Note that the p-values for the comparisons of the cumulative total number of inbound and outbound trials are larger than those derived from the non-parametric repeated measures test presented in the main text, because the repeated measures test takes into account the day-by-day trend for each individual subject.
(0.02 MB PDF)

Figure S1 Moving-average learning curves for individual control subjects on the W-track continuous alternation task. Each panel shows 10-trial moving averages of task performance for one control animal. The top plot in each panel shows performance on inbound trials, while the bottom plot shows performance on outbound trials. Trials are counted cumulatively along the horizontal axis, starting with the 10th trial on day 1 and ending with the last trial on day 10. The alternating blue and green background shading indicates the number of trials completed on each day.
(0.86 MB TIF)

Figure S2 Moving-average learning curves for individual hippocampal lesion subjects on the W-track continuous alternation task. Each panel shows 10-trial moving averages of task performance for one lesion animal. The top plot in each panel shows performance on inbound trials, while the bottom plot shows performance on outbound trials. Trials are counted cumulatively along the horizontal axis, starting with the 10th trial on day 1 and ending with the last trial on day 10. The alternating blue and green background shading indicates the number of trials completed on each day.
(1.54 MB TIF)

Figure S3 Smooth learning curves for individual control subjects on the W-track continuous alternation task. Each panel shows the estimated probability of correct performance for one control animal. The top plot in each panel shows the estimated learning curve for the inbound component of the task, while the bottom plot shows the estimated learning curve for the outbound component of the task. Trials are counted cumulatively along the horizontal axis, starting with the first trial on day 1 and ending with the last trial on day 10. The alternating blue and green background shading indicates the number of trials completed on each day. Black dots indicate maximum-likelihood estimates of the probability of correct performance, and gray errors bars indicate point-wise 95% confidence intervals. Dashed horizontal lines indicate the chance performance level (1/2) that would be expected if subjects randomly chose the destination food well on each trial. We defined the learning criterion (highlighted in red) as the trial on which the 95% confidence interval of the learning curve exceeded this chance level and thereafter remained above chance throughout two full consecutive days of testing.
(0.67 MB TIF)

Figure S4 Smooth learning curves for individual hippocampal lesion subjects on the W-track continuous alternation task. Each panel shows the estimated probability of correct performance for one lesion animal. For explanation, see the legend for Figure S3. Lesioned subjects were much more variable in their task performance than subjects in the control group. They often performed below chance level on the inbound component of the task during the first few days, reflecting perseverative errors (see Figure 6). By our learning criterion, three of the six lesion subjects failed to learn the outbound component of the task by the end of testing.
(1.14 MB TIF)
