# Supplementary material for: Hippocampal Lesions Impair Rapid Learning of a Continuous Spatial Alternation Task
Source: PLoS One. 2009 May 8;4(5):e5494. doi: 10.1371/journal.pone.0005494 (PMC2674562; doi:10.1371/journal.pone.0005494)
Supplement: Table S1 — Summary statistics for individual subjects on the W-track continuous alternation task. Each column corresponds to an individual subject; C1-C4 are control subjects, and L1-L6 are hippocampal lesion subjects. The p-value column shows the result of the Wilcoxon rank-sum comparison between the two groups. Note that the p-values for the comparisons of the cumulative total number of inbound and outbound trials are larger than those derived from the non-parametric repeated measures test presented in the main text, because the repeated measures test takes into account the day-by-day trend for each individual subject. (0.02 MB PDF) [file pone.0005494.s001.pdf]

Table S1. Measures of behavioral performance for each animal.

		Control subjects				Hippocampal lesion subjects						*p*-value of between-groups comparison (Wilcoxon rank sum test)
		C1	C2	C3	C4	L1	L2	L3	L4	L5	L6	
Inbound	Total number of inbound trials performed, days 1-10	576	493	489	650	481	810	996	736	1064	1036	0.11
Inbound	Number of inbound trials to reach learning criterion on inbound task component	266	150	43	77	297	778	124	>736*	819	668	0.038
Inbound	Days to reach learning criterion on inbound task component	6	4	2	2	8	10	2	>10*	8	7	0.057
Inbound	Mean estimated probability of correct inbound performance on day 10	0.96	0.98	0.94	0.97	0.95	0.77	0.92	0.70	0.89	1.00	0.26
Outbound	Total number of outbound trials performed, days 1-10	519	259	535	587	269	602	856	594	762	847	0.11
Outbound	Number of outbound trials to reach learning criterion on inbound task component	55	214	37	58	>269*	>602*	381	483	410	>847*	0.0095
Outbound	Days to reach learning criterion on outbound task component	3	5	2	2	>10*	>10*	6	9	7	>10*	0.0095
Outbound	Mean estimated probability of correct outbound performance on day 10	0.85	0.93	0.84	0.87	0.14	0.44	0.96	0.85	0.79	0.84	0.17

*Asterisks indicate that the subject did not reach the learning criterion within the 10 days of testing.
