# Supplementary figures and images for: Hippocampal Lesions Impair Rapid Learning of a Continuous Spatial Alternation Task
Source: PLoS One. 2009 May 8;4(5):e5494. doi: 10.1371/journal.pone.0005494 (PMC2674562; doi:10.1371/journal.pone.0005494)

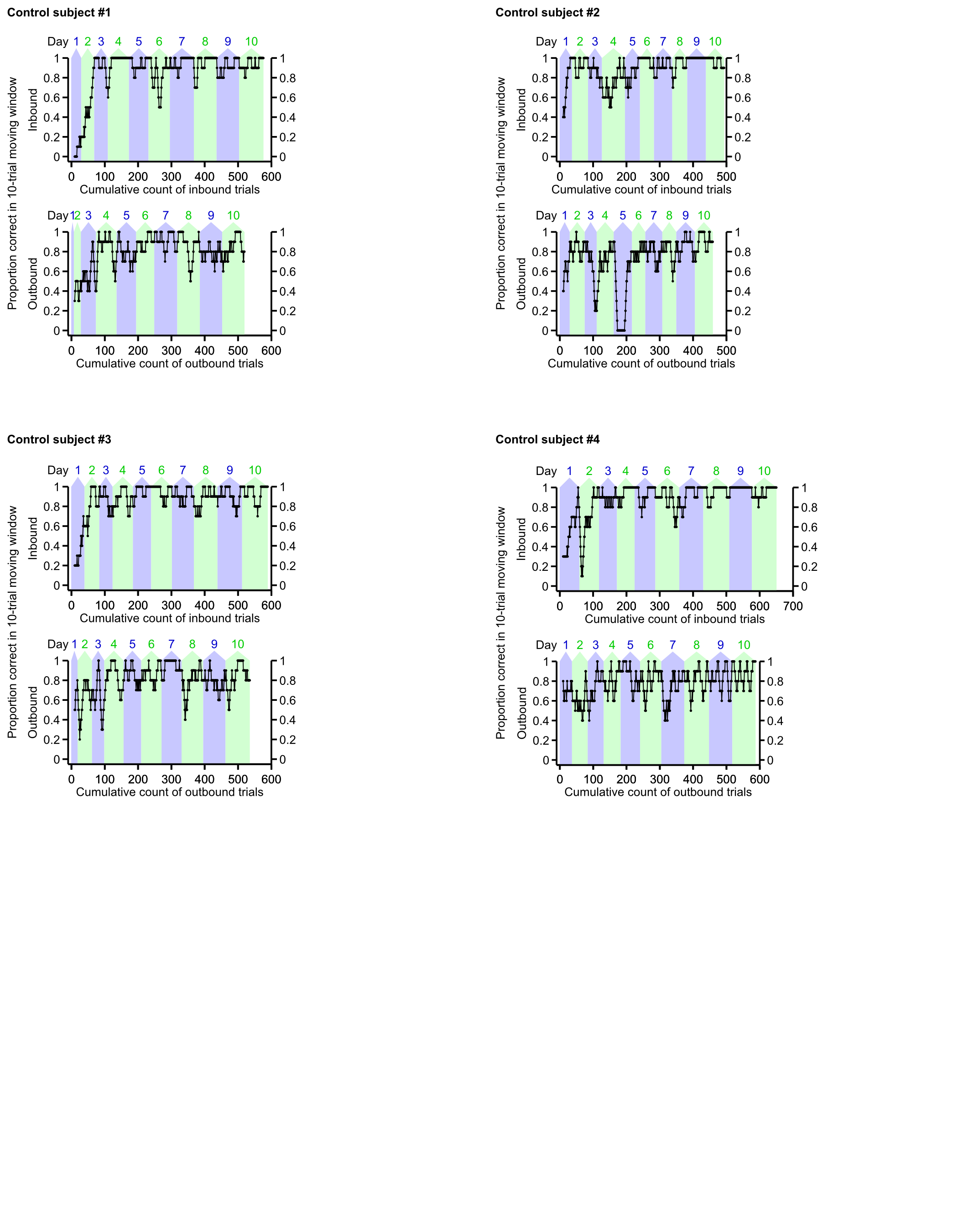

Supplement: Figure S1 — Moving-average learning curves for individual control subjects on the W-track continuous alternation task. Each panel shows 10-trial moving averages of task performance for one control animal. The top plot in each panel shows performance on inbound trials, while the bottom plot shows performance on outbound trials. Trials are counted cumulatively along the horizontal axis, starting with the 10th trial on day 1 and ending with the last trial on day 10. The alternating blue and green background shading indicates the number of trials completed on each day. (0.86 MB TIF) [file pone.0005494.s002.tif]

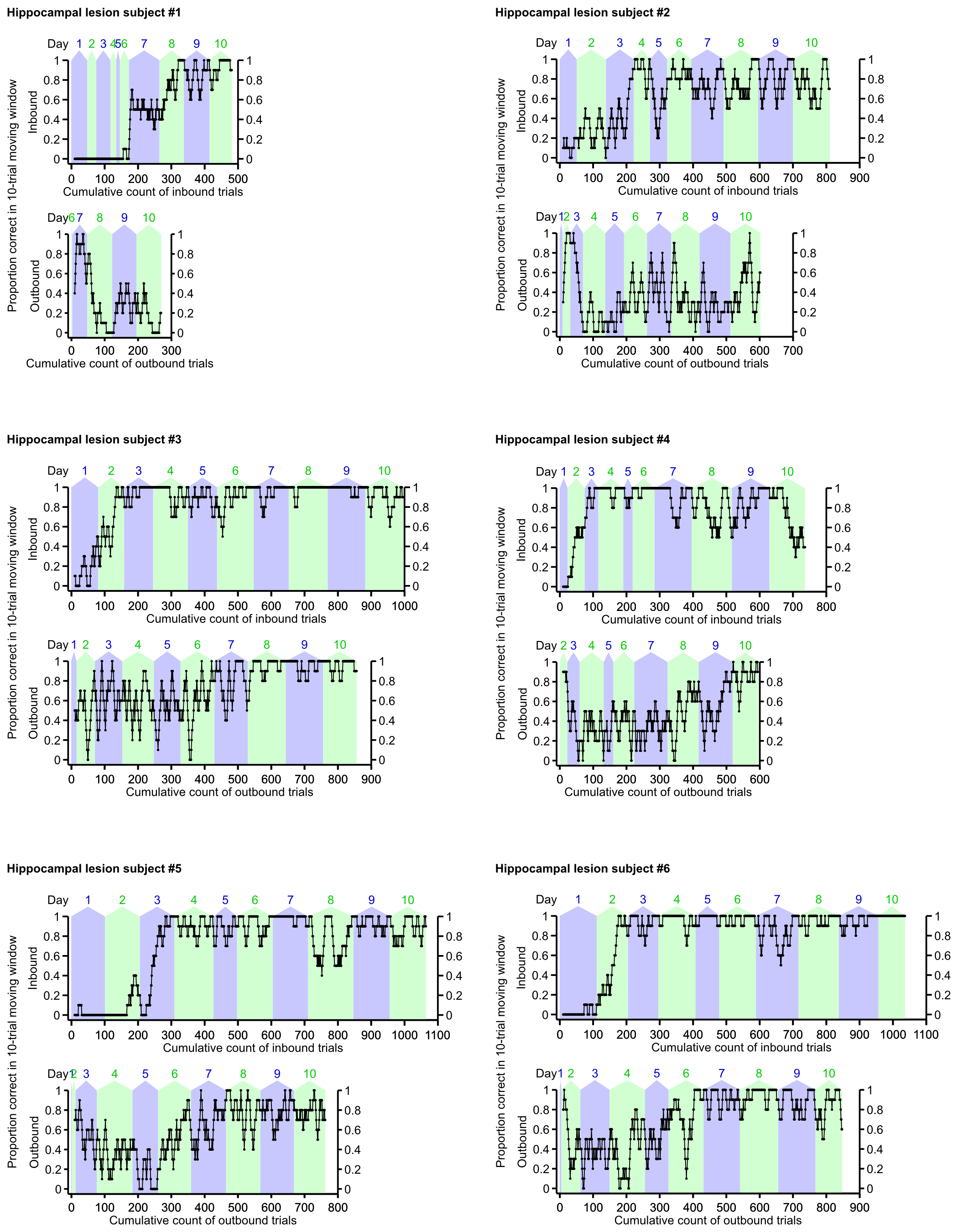

Supplement: Figure S2 — Moving-average learning curves for individual hippocampal lesion subjects on the W-track continuous alternation task. Each panel shows 10-trial moving averages of task performance for one lesion animal. The top plot in each panel shows performance on inbound trials, while the bottom plot shows performance on outbound trials. Trials are counted cumulatively along the horizontal axis, starting with the 10th trial on day 1 and ending with the last trial on day 10. The alternating blue and green background shading indicates the number of trials completed on each day. (1.54 MB TIF) [file pone.0005494.s003.tif]

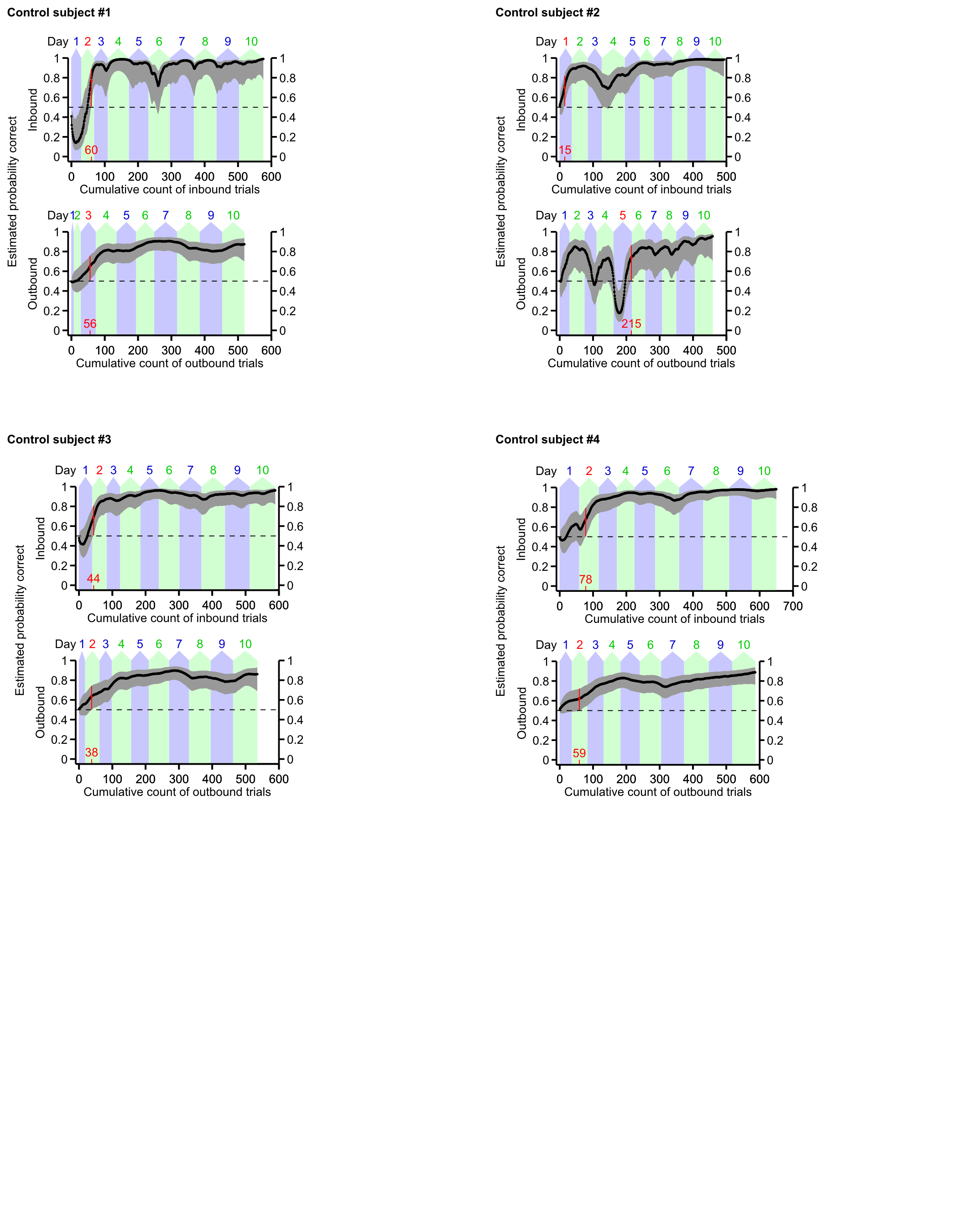

Supplement: Figure S3 — Smooth learning curves for individual control subjects on the W-track continuous alternation task. Each panel shows the estimated probability of correct performance for one control animal. The top plot in each panel shows the estimated learning curve for the inbound component of the task, while the bottom plot shows the estimated learning curve for the outbound component of the task. Trials are counted cumulatively along the horizontal axis, starting with the first trial on day 1 and ending with the last trial on day 10. The alternating blue and green background shading indicates the number of trials completed on each day. Black dots indicate maximum-likelihood estimates of the probability of correct performance, and gray errors bars indicate point-wise 95% confidence intervals. Dashed horizontal lines indicate the chance performance level (1/2) that would be expected if subjects randomly chose the destination food well on each trial. We defined the learning criterion (highlighted in red) as the trial on which the 95% confidence interval of the learning curve exceeded this chance level and thereafter remained above chance throughout two full consecutive days of testing. (0.67 MB TIF) [file pone.0005494.s004.tif]

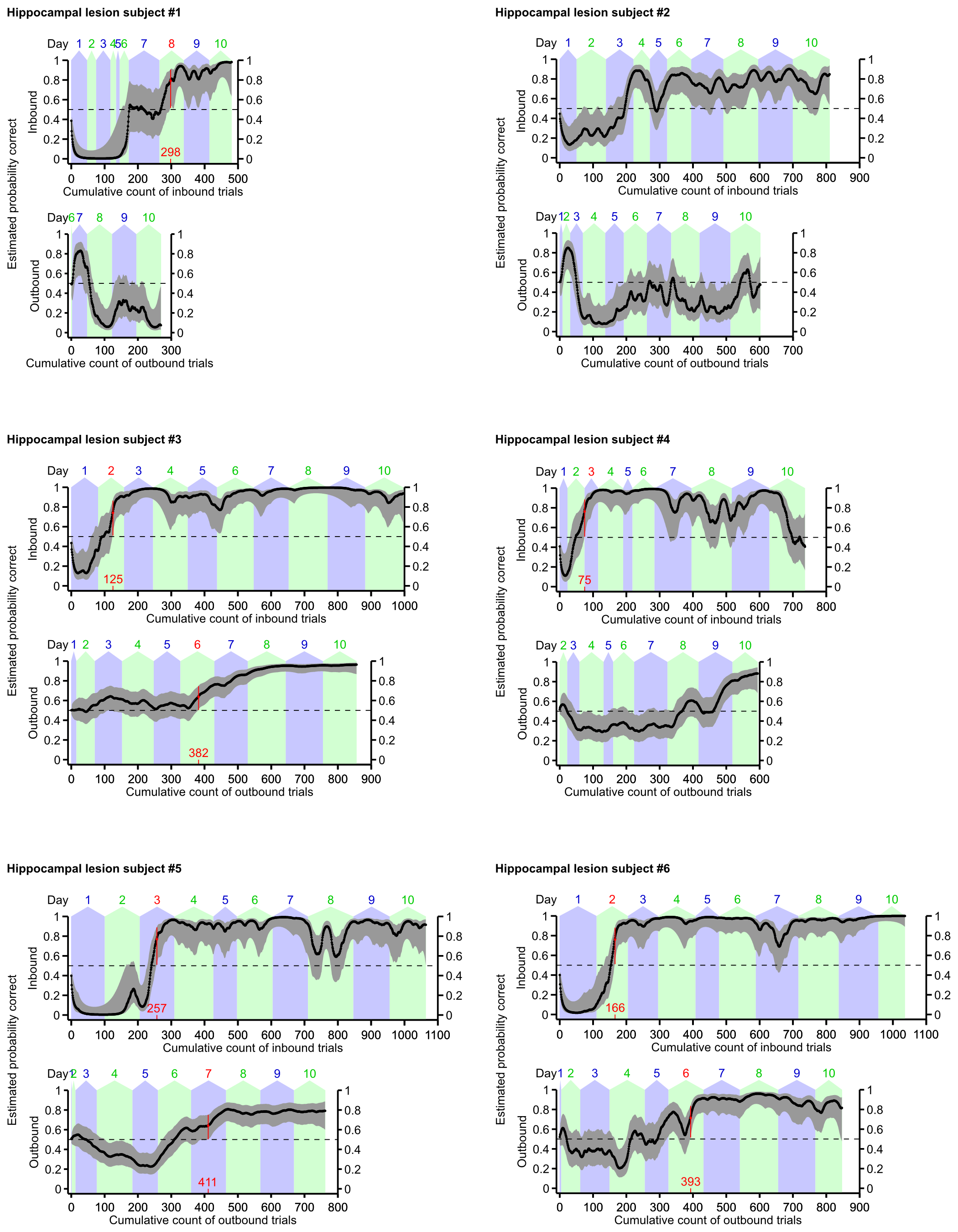

Supplement: Figure S4 — Smooth learning curves for individual hippocampal lesion subjects on the W-track continuous alternation task. Each panel shows the estimated probability of correct performance for one lesion animal. For explanation, see the legend for Figure S3. Lesioned subjects were much more variable in their task performance than subjects in the control group. They often performed below chance level on the inbound component of the task during the first few days, reflecting perseverative errors (see Figure 6). By our learning criterion, three of the six lesion subjects failed to learn the outbound component of the task by the end of testing. (1.14 MB TIF) [file pone.0005494.s005.tif]
